# Effects of Implant Silver Coatings on Bone Formation in Animal Models: A Systematic Review and Meta-Analysis

**DOI:** 10.3390/jfb16100369

**Published:** 2025-10-01

**Authors:** Ali Alenezi

**Affiliations:** Department of Prosthetic Dental Sciences, College of Dentistry, Qassim University, Buraydah 51452, Saudi Arabia; ali.alenezi@qu.edu.sa; Tel.: +966-163800050 (ext. 2039)

**Keywords:** silver-coated titanium implants, bone formation, peri-implantitis, antibacterial coatings, animal models, meta-analysis

## Abstract

Background/Objective: Clinical statistics show that bacterial infection is a major driver of implant failure. To enhance antibacterial performance, some metallic elements, such as silver (Ag), zinc (Zn), and copper (Cu), are commonly used to modify the titanium surface. Despite the promising antibacterial performance of Ag, concerns persist regarding dose-dependent cytotoxicity, systemic accumulation, and potential effects on local bone metabolism. This review aimed to investigate the effects of incorporating or coating titanium (Ti) implant surfaces with Ag on bone formation around implants. Methods: A search was undertaken using three main databases (PubMed, Web of Science, and Scopus). The search was limited to studies published within the last 20 years that involved animal experiments using endosseous implants coated with or incorporating Ag. Meta-analyses were performed for bone-to-implant contact (BIC), bone formation (BA), and bone volume (BV/TV) around the implant in control and test groups. The compared groups were subjected to similar implant surface treatments aside from the presence of silver in the test group. Results: Sixteen studies met the inclusion criteria in this study and were included. The analysis of BIC values revealed a statistically significant overall effect in favor of silver-coated implants (Z = 2.01, *p* = 0.04), along with 95% confidence intervals (CIs). The BA analysis found no significant difference between silver-coated and control implants (Z = 1.09, *p* = 0.28). The BV/TV analysis also showed no statistically significant overall difference (Z = 0.35, *p* = 0.73). Conclusions: In animal models, silver-coated Ti implants improve bone–implant contact without altering peri-implant bone volume metrics.

## 1. Introduction

Titanium (Ti) and its alloys are regularly used in implantable medical and dental devices because of their favorable biocompatibility, mechanical strength, and corrosion resistance [1,2]. Ti implants now represent the preferred modality for the restoration of edentulous spaces in dental practice [3,4]. Osseointegration around titanium (Ti) implants is a coordinated cascade of immune, angiogenic, and osteogenic events shaped by surgical protocol, implant surface chemistry/topography, primary stability, functional loading, and host factors [5,6]. However, unlike many other surgical procedures performed under strict aseptic conditions, dFental implants are immediately exposed to the complex oral environment, which is home to diverse microorganisms [7,8].

Peri-implant infections are among the most significant complications related to dental implants, which involve inflammation of peri-implant tissues and continuing marginal bone loss [3,9]. These problems are more pronounced in individuals whose bone healing is compromised by diabetes, older age, or osteoporosis [10,11]. Numerous studies have demonstrated a strong association between peri-implant infections and implant failure, with microbial biofilms playing a pivotal role in disease initiation and progression [6,10]. Once an implant-related infection is established, treatment often requires surgical debridement, implant removal, prolonged antibiotic therapy and, eventually, revision surgery, which imposes substantial clinical and financial burdens on both patients and healthcare systems [10,12].

To reduce such risks, several studies have focused on modifying implant surfaces to enhance osseointegration while minimizing bacterial colonization [13,14]. Nanoscale or microscale roughness could improve the adhesion and proliferation of osteoblasts, accelerating bone healing [13,15]. However, rougher surfaces at exposed regions can also promote bacterial attachment, potentially increasing the risk of infection [16,17]. Coating Ti implants with antimicrobial agents is one strategy to address this issue. Such coatings aim to provide an initial antibacterial effect during the early healing phase, when infection risk is highest, while maintaining compatibility with host tissues [18].

Silver (Ag) is one of the most extensively investigated antimicrobial agents for implant coatings [19,20]. It is active against a wide spectrum of pathogens, including many multidrug-resistant isolates, and its bactericidal properties have been attributed to multiple mechanisms: bacterial membrane disruption, reactive oxygen species generation, and inhibition of essential enzymatic systems [21,22]. Silver nanoparticles (Ag NPs) have enhanced antimicrobial activity compared with bulk Ag, mainly due to their large specific surface area and continuous release of Ag^+^ ions [20,23].

In vitro and in vivo reports have revealed the ability of Ag-coated Ti surfaces to reduce bacterial adhesion and biofilm formation without significantly impairing osteoblast function when used at controlled concentrations [24,25]. For example, Ti implants modified with Ag NPs have shown significant antibacterial effects against Staphylococcus aureus while maintaining favorable cell viability and alkaline phosphatase activity in osteoblast cultures [26]. Preclinical models have also reported reduced peri-implant infection with Ag-coated Ti implants and bone–implant contact ratios comparable to those of uncoated controls [27,28].

Several methods have been investigated to incorporate Ag into Ti implant surfaces, including plasma-immersion ion implantation, electrochemical deposition, and sol–gel techniques. Each method influences the release kinetics of Ag ions, which is critical for balancing antimicrobial efficacy with cytocompatibility [29,30]. Controlled, low-level Ag release is particularly desirable to minimize cytotoxic effects on host cells, as high local concentrations can impair osteoblast proliferation and differentiation [31,32].

The clinical use of Ag-coated Ti implants in orthopedic applications is more established, with Ag-coated prostheses having been successfully used in tumor surgery to prevent deep infections [33,34]. In dentistry, however, the clinical data remain limited. Early-stage studies have suggested that Ag coatings can reduce early colonization by pathogenic bacteria, but the long-term effects on bone remodeling and implant survival remain unclear [35]. Despite the promising antibacterial performance of Ag, concerns persist regarding its potential cytotoxicity, systemic accumulation, and the possibility of altering local bone metabolism. Some studies have reported delayed bone healing or reduced bone–implant contact at higher Ag concentrations, highlighting the need for precise control over release profiles [36,37]. The cytotoxicity of AgNPs is dose- and time-dependent [38]; however, at appropriately low concentrations, AgNPs can enhance cell proliferation and activity [39].

Given these considerations, this review investigated the effects of incorporating or coating Ti implant surfaces with Ag on bone formation around implants and whether antibacterial benefits can be achieved without compromising osseointegration.

## 2. Materials and Methods

### 2.1. PICO Question

This review was performed in accordance with the PRISMA guidelines (Preferred Reporting Items for Systematic Reviews and Meta-Analyses, INPLASY registration number: INPLASY202590035) [40]. Before initiating the systematic literature search, the PICO framework was defined as follows: the Population (*p*) was defined as animal models; the Intervention (I) was Ti implants modified or coated with silver; the Control (C) was defined as Ti implants without silver modifications or coatings; and the Outcome (O) was bone formation.

### 2.2. Search Strategies

The literature search was undertaken in May 2025 using three main databases: PubMed, Web of Science, and Scopus. Only studies published within the last 20 years were considered. The search strategies in all the databases included terms related to four primary aspects: bone, implant, silver, and animal studies. The search terms used for the electronic search were (“bone formation” OR “osseointegration” OR “bone growth” OR “bone development” OR “bone remodeling” OR “bone regeneration”) AND (“implants” OR “titanium implants” OR “dental implants” OR “biocompatible coated materials” OR “endosseous dental implantation” OR “bone-implant interface”) AND (“antibacterial” OR “antimicrobial” OR “infection” OR “failure” OR “Coatings” OR “silver” OR “silver nanoparticles” OR “silver coating” OR “Ag”) AND (“in vivo” OR “animals” OR “animal models” OR “animal experimentation”).

### 2.3. Inclusion and Exclusion Criteria

The eligibility criteria were (1) studies conducted on animals that evaluated the use of silver coatings with endosseous implants. (2) The silver should be incorporated into the substrate or applied as a surface coating for these implants, either prior to or at the time of insertion. (3) Measurements of bone–implant contact (BIC), bone area (BA), or bone volume (BV/TV) should be reported for the control and test groups. (4) The compared groups should have received similar implant surface treatments aside from the presence of silver in the test group. (5) Only articles published in English were included. Clinical studies or in vitro investigations were excluded. Studies that did not report BIC, BA, or BV/TV values and the number of implants used in the evaluated groups were excluded.

### 2.4. Study Selection

All studies identified in the initial search were screened for eligibility according to the prespecified inclusion criteria. Full-text retrieval was performed for studies that fulfilled these criteria and those whose titles/abstracts lacked sufficient detail to determine their eligibility.

### 2.5. Data Extraction

When available, the following variables were collected via a standardized extraction form: study title, publication year, coating method, form of silver, animal species, number of animals and implants, duration between implant placement and animal euthanasia, as well as the mean values and standard deviations for BIC, BA, and BV/TV percentages around the implants.

### 2.6. Quality Assessment

The methodological quality was evaluated using the ARRIVE 2.0 (Animal Research: Reporting of In Vivo Experiments) guidelines [41]. Each of the 21 items was categorized as follows: “reported” (two points) if all subitems were fully addressed, “not reported” (no points) if none were addressed, and “unclear” (one point) if some details were missing. Based on these scores, the quality coefficient was calculated for each study, which was categorized as follows: Poor (<0.5), Average (0.5–0.8), or Excellent (0.8–1).

### 2.7. Risk of Bias in Individual Studies

The risk of bias was estimated using the SYRCLE (Systematic Review Centre for Laboratory Animal Experimentation) RoB tool [42].

### 2.8. Data Analysis

The continuous outcomes assessed were BIC, BA, and BV/TV. The weighted mean differences were used to generate forest plots, with the number of implants in each group serving as the statistical unit. Records with insufficiently reported outcomes of interest were excluded from the analysis. Heterogeneity between the studies was quantified using the I^2^ statistic, which represents the percentage of total variation due to heterogeneity. When statistical heterogeneity reached significance (*p* < 0.05), a random-effects (inverse-variance) model was used; if not, a fixed-effect model was used. Intervention effects are reported as mean differences (MDs) in percentage, along with 95% confidence intervals (CIs). The meta-analysis was completed using the Review Manager software (version 5.3.3, The Nordic Cochrane Centre, The Cochrane Collaboration).

## 3. Results

### 3.1. Literature Search

**Figure 1:**
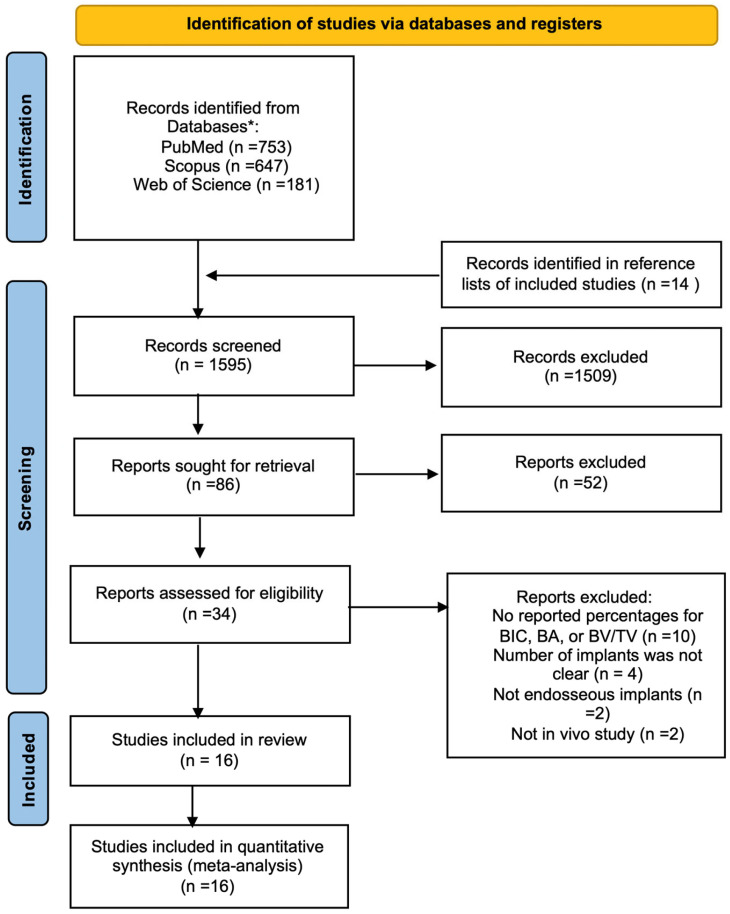
PRISMA flow diagram for the search process to identify studies to include in this review. The “*” represent the three main databases used for the electronic search: PubMed, Web of Science, and Scopus.

Figure 1 shows an overview of the study selection process. Following database searching, screening of titles and abstracts, and removal of duplicates, the full-text articles were evaluated for eligibility. In total, 16 animal studies met the eligibility criteria and were included in both the qualitative and quantitative syntheses [28,43,44,45,46,47,48,49,50,51,52,53,54,55,56,57]. PRISMA checklist is shown in Supplementary Materials (Table S1).

The study characteristics of the included articles are shown in Table 1. The investigations were conducted in a range of animal models including Wistar rats, New Zealand White rabbits, Sprague–Dawley rats, Labrador dogs, beagle dogs, pigs, and Merino sheep. A variety of silver modification strategies were reported, including nanotubular structures, plasma immersion ion implantation (PIII), electrodeposition, atomic layer deposition, wet chemical plating, magnetron sputtering, and hybrid coatings. Silver was delivered in several forms, such as nanoparticles, ions, nanolayers, and silver oxide. The outcome assessment methods predominantly employed micro-computed tomography (micro-CT) and histomorphometric analysis.

### 3.2. Quality Assessment

**Figure 2:**
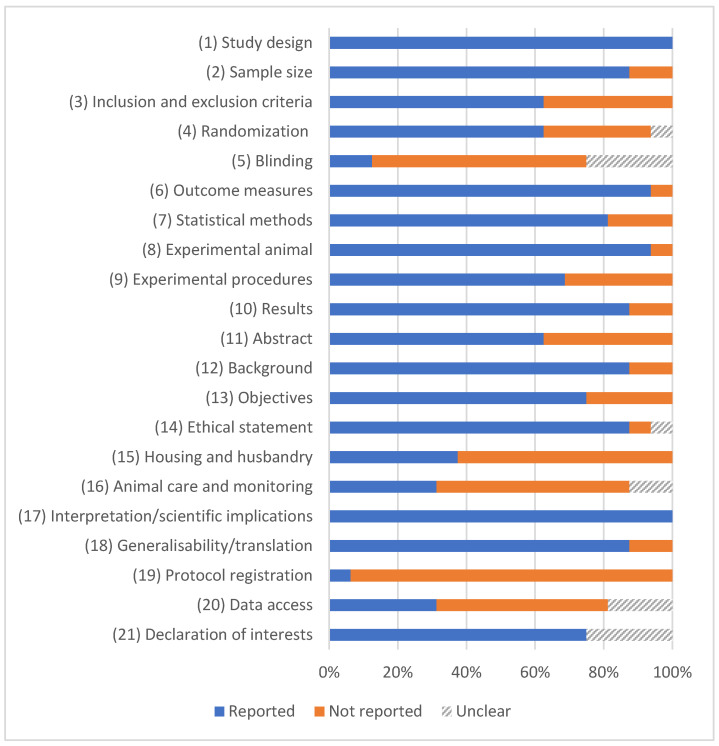
ARRIVE 2.0-based quality evaluation of included studies. Values are presented as percentages.

Quality was assessed using the ARRIVE 2.0 guidelines and the results are presented in Figure 2 and Table 2. The calculated coefficients ranged between 0.71 and 0.93. Eight studies achieved a rating of Excellent [28,44,45,48,50,52,53,56], while the remaining eight were graded as Average. No study was categorized as Poor.

### 3.3. Risk of Bias

**Figure 3:**
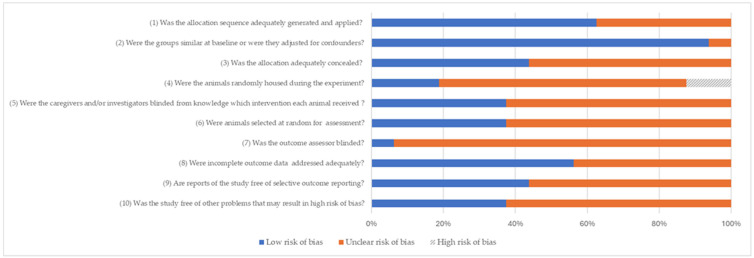
Risk of bias distribution according to Systematic Review Centre for Laboratory Animal Experimentation (SYRCLE) tool. Values are presented as percentages.

The assessment of the risk of bias based on the SYRCLE guide is displayed in Figure 3. In most studies, the risk of bias was judged to be unclear for both allocation concealment and outcome assessor blinding. Domains related to random sequence generation and selective outcome reporting showed lower levels of concern. Overall, variability in methodological care was observed among the included studies.

### 3.4. Meta-Analysis of Bone Formation Outcomes

#### 3.4.1. BIC

**Figure 4:**
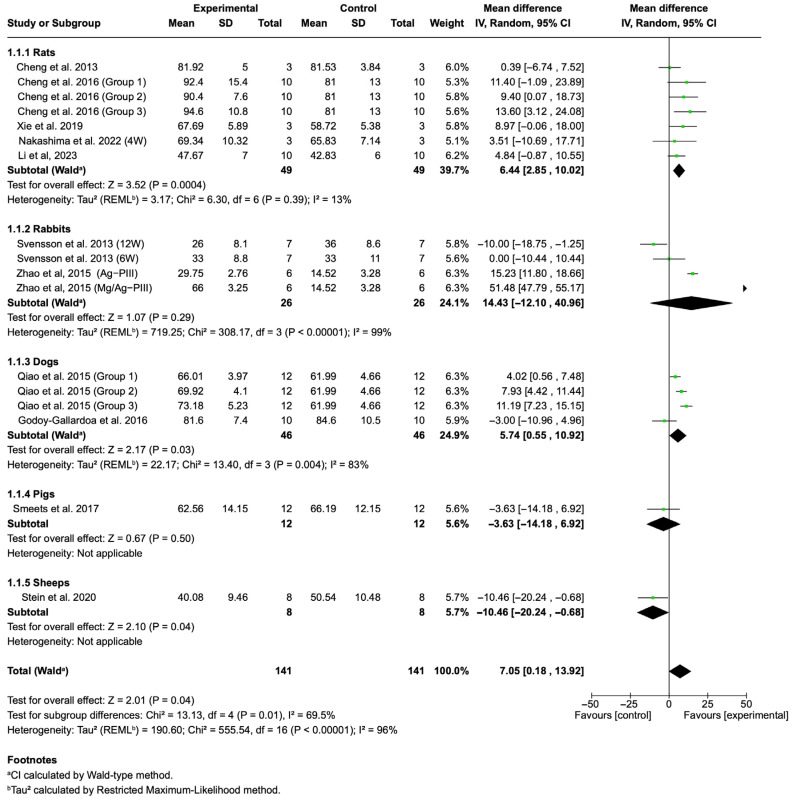
Forest plot summarizing the BIC values from the included studies on Ti implants with silver coatings [28,43,44,45,47,48,50,52,53,56]. The pooled analysis revealed a statistically significant overall effect in favor of silver-coated implants (Z = 2.01, *p* = 0.04).

The pooled analysis of BIC values (Figure 4) revealed a statistically significant overall effect in favor of silver-coated implants (Z = 2.01, *p* = 0.04). Substantial heterogeneity was observed (Tau^2^ = 190.60; Chi^2^ = 555.54, df = 16 (*p* < 0.001); I^2^ = 96%).

#### 3.4.2. BA

**Figure 5:**
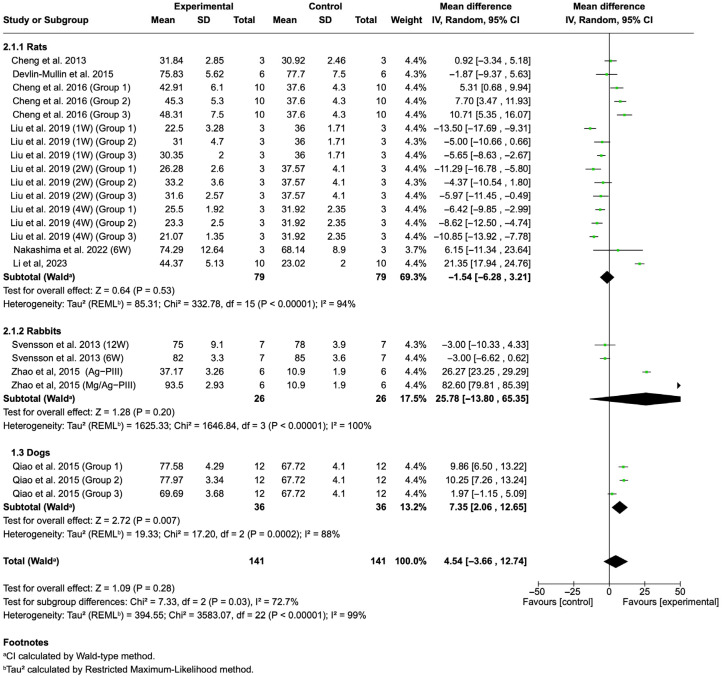
Forest plot summarizing the BA values from the included studies on Ti implants with silver coatings [28,43,44,45,46,47,49,51,56]. The pooled analysis demonstrated no significant difference between silver-coated and control implants (Z = 1.09, *p =* 0.28).

The pooled analysis of BA (Figure 5) demonstrated no significant difference between silver-coated and control implants (Z = 1.09, *p* = 0.28). Considerable heterogeneity was observed (Tau^2^ = 394.55; Chi^2^ = 3583.07, df = 22 (*p* < 0.001); I^2^ = 99%).

#### 3.4.3. BV/TV

**Figure 6:**
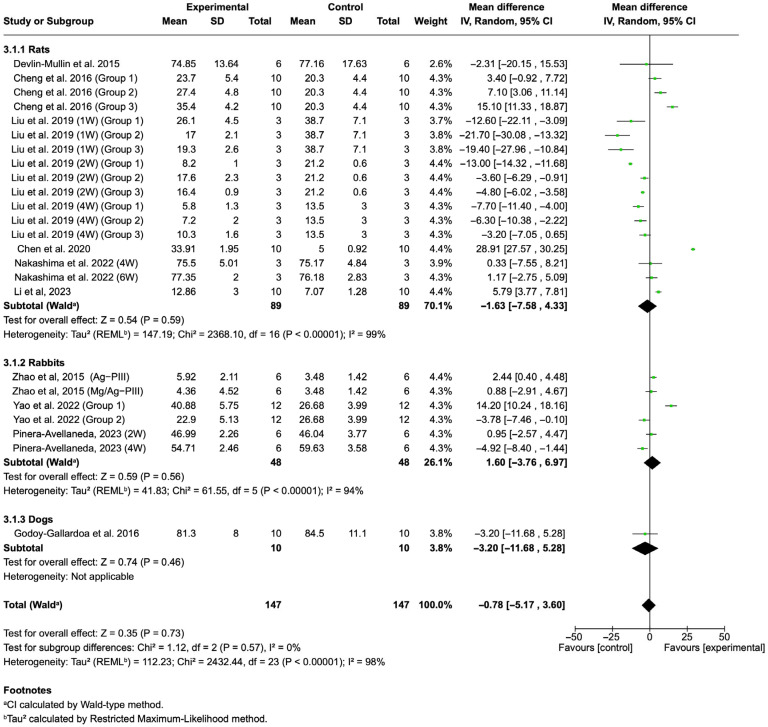
Forest plot summarizing the BV/TV values from the included studies on Ti implants with silver coatings [28,46,47,48,49,51,54,55,56,57]. The analysis of BV/TV showed no statistically significant overall difference (Z = 0.35, *p* = 0.73).

The analysis of BV/TV (Figure 6) also showed no statistically significant overall difference (Z = 0.35, *p* = 0.73). High heterogeneity was detected across studies (Tau^2^ = 112.23; Chi^2^ = 2432.44, df = 23 (*p* < 0.001); I^2^ = 98%).

## 4. Discussion

This review evaluated the effect of Ag-coated Ti implants on bone formation around implant in animal models. Sixteen studies were included, and the pooled findings revealed that Ag coatings significantly improved BIC, while no significant differences were found for BA or BV/TV. These results indicate that Ag modifications may facilitate bone integration but do not consistently enhance overall peri-implant bone remodeling.

The integration of Ag into dental implant surfaces has been primarily driven by its antimicrobial potential, as peri-implant infections are among the most prevalent reasons for implant loss. Peri-implantitis is initiated by bacterial colonization and biofilm formation on implant surfaces, leading to a cascade of host immune responses, chronic inflammation, and progressive marginal bone loss [58,59]. Therefore, antibacterial surface modifications are being increasingly explored to prevent early microbial colonization and reduce the incidence of biological complications. Among these modifications, Ag has emerged as a particularly attractive candidate because of its broad-spectrum antibacterial ability and relatively low tendency to develop resistance [19,60].

At the molecular level, Ag ions exert bactericidal effects by binding to bacterial cell membranes, increasing membrane permeability, denaturing enzymes, and interfering with DNA replication [61]. Nanosilver particles also generate reactive oxygen species, further enhancing their antimicrobial efficacy. This potent antibacterial activity has been observed both in vitro and in vivo, with Ag-coated implants showing significantly reduced bacterial adhesion compared with unmodified Ti implants [35,62]. In the orthopedic field, clinical studies have confirmed reduced infection rates with Ag-coated prostheses [63,64] while dental research has focused on whether similar benefits can be achieved in oral implantology. Beyond antibacterial effects, Ti surfaces modified with Ag can promote early macrophage polarization from a pro-inflammatory M1 state to a pro-healing M2 phenotype, thereby moderating inflammation and supporting osteogenesis [54]. In parallel, sustained Ag release is associated with pro-angiogenic signaling, enhanced endothelial tube formation, and an increased microvessel density, collectively strengthening the angiogenesis–osteogenesis coupling required for durable osseointegration [24].

The significant improvement in BIC found in this review suggests that Ag coatings may support early osseointegration, either directly by enhancing osteoblast behavior or indirectly by suppressing bacterial competition at the interface (Figure 4). Several in vivo studies have confirmed this effect. For instance, Cheng et al. [49] demonstrated that Ag NP-modified nanotubular implants promoted greater BIC in rat models, while Xie et al. [52] observed improved osseointegration with Ag–hydroxyapatite hybrid coatings. At the cellular level, Ag has been reported to stimulate osteoblast adhesion and proliferation at low concentrations [35,65]. Furthermore, recent evidence has suggested that Ag coatings may also influence immune modulation, promoting a favorable M2 macrophage phenotype that enhances osteogenesis and angiogenesis [30,66]. This dual antibacterial–immunomodulatory function may explain why Ag coatings improved BIC across diverse experimental conditions.

In contrast, no significant differences were observed in BA or BV/TV, which is a volumetric indicator of peri-implant bone formation (Figure 4 and Figure 5). This discrepancy between interfacial contact and bone volume suggests that the effect of Ag may be localized to the implant–bone interface rather than extending to overall bone remodeling. Several factors may account for this variability. First, the dose-dependent effects of Ag are critical: while low concentrations are osteoconductive, higher concentrations may impair osteoblast proliferation, collagen synthesis, and matrix mineralization [31,38]. Zhao et al. [35] demonstrated that excessive nanosilver exposure reduced osteoblast activity, while Greulich et al. [67] showed cytotoxicity to bone marrow stromal cells at elevated Ag doses. Second, discrepancies in coating techniques (e.g., plasma immersion ion implantation, electrodeposition, magnetron sputtering, and atomic layer deposition) lead to differences in Ag release profiles, which strongly influence biological outcomes [35,50]. Third, most studies used short- or medium-term follow-up, which may not capture the long-term remodeling processes critical for implant survival.

These findings position Ag coatings as dual-function surfaces in implant dentistry: their primary role remains antibacterial, protecting against peri-implant infections, while also appearing to maintain or even enhance interfacial bone contact. More importantly, none of the included studies in this review reported detrimental effects on bone formation, addressing the concerns about the potential cytotoxicity of Ag. This is reassuring for clinical translation, as infection control and bone preservation are interdependent requirements for long-term implant success. Notably, peri-implantitis and marginal bone loss often coexist, particularly in patients at high risk such as those with diabetes, poor oral hygiene, or osteoporosis [10,11,68]. For these populations, Ag-coated implants could offer a valuable adjunctive strategy to reduce complications.

When comparing Ag with other antimicrobial coatings, such as zinc, copper, or chitosan, the evidence suggests that while they all provide antibacterial activity, Ag remains the most extensively studied and exhibits one of the broadest spectrum of antimicrobial efficacy [35,66]. However, other materials may provide additional osteogenic benefits [69]. Zinc (Zn) coatings on titanium implants exhibit favorable bactericidal effects attributable to Zn incorporation [70], and in vivo studies suggest bacteria show limited resistance to Zn^2+^ [71]. Zn has also been associated with enhanced osteoblastic activity, supporting its use in bone-related applications [72,73]. Copper (Cu) can provide broad-spectrum antimicrobial activity [74]. Furthermore, Cu can be integrated into implant alloys such as Ti-6Al-4V to strengthen antibacterial performance and to enhance angiogenesis [75,76]. However, unlike Ag, elevated Copper ions (Cu^2+^) release has been linked to cytotoxicity, which necessitate careful control of release kinetics and coating thickness [35,77]. Comparative research is needed to determine whether Ag provides superior clinical benefits or whether combinations of antimicrobial and osteoinductive coatings may represent the optimal approach.

The limitations of the available evidence must be acknowledged. Large discrepancies were observed in all the pooled outcomes, reflecting the differences in animal species, sample sizes, healing times, and methodological approaches (Table 1). The risk of bias was often unclear due to inadequate reporting of allocation concealment and blinding (Figure 3). Moreover, peri-implantitis and marginal bone loss are long-term complications, but the included studies largely reported short-term results (≤6 months). Thus, it remains uncertain whether the antibacterial advantages of Ag coatings translate into sustained improvements in peri-implant bone stability and implant survival.

Future research should prioritize long-term preclinical studies using standardized protocols and large animal models to better approximate clinical conditions. Optimization of Ag concentration, particle size, and release kinetics is necessary to balance antibacterial efficacy with cytocompatibility. Moreover, comparative clinical trials are needed to assess Ag’s effectiveness relative to other antibacterial coatings and to confirm whether the observed improvements in BIC can be translated into reduced risks of peri-implant disease and bone loss in patients.

## 5. Conclusions

This review demonstrated that Ag-coated Ti implants are associated with a significant improvement in BIC, whereas no consistent effects were observed for BA or BV/TV. These findings suggest that Ag coatings, while primarily applied for their antibacterial properties, may also support interfacial bone healing without compromising peri-implant bone formation. Further preclinical and clinical investigations are warranted to clarify the dose–response relationship, optimize coating strategies, and determine whether the antibacterial advantages of Ag coatings translate into improved peri-implant bone preservation and implant survival in dental practice.

## Figures and Tables

**Table 1:** Overview of characteristics of included studies.

Author	Silver Coating Technique	Silver Form	Animal Model	Bone Formation Outcome(s)	Evaluation Method(s)	Evaluated Groups	Healing Period
Cheng, 2013 [43]	Silver nanoparticle (Ag NPs) incorporated nanostructured Ti coating	Ag NPs	SD rats	BIC and BA	Histological evaluation	Control: Ti oxide nanotubes (TiO_2_-NT); test group: NT-Ag	8 weeks
Svensson, 2013 [44]	Wet chemical plating process	Nanostructured coating	New Zealand White rabbits	BIC and BA	Histological evaluation	Control: non-coated implants; test group: coated implants	6 and 12 weeks
Qiao, 2015 [45]	Plasma immersion ion implantation of silver (Ag-PIII)	Ag NPs	Labrador dogs	BA and BIC	Micro-CT and histological evaluation	Control: sandblasted and acid-etched (SLA) implants; test groups: implants treated for (1) 30 min with 30 Ag PIII, (2) 60 min with 30 Ag PIII, and (3) 90 min with 30 Ag PIII	3 months
Devlin-Mullin, 2015 [46]	Silver nanolayer deposition via atomic layer deposition	Ag Nanolayer	Wistar rats	BA	Micro-CT analysis	Control: Ti; test group: Ti-Ag	12 weeks
Zhao, 2015 [47]	Plasma immersion ion implantation of silver (Ag-PIII)	Ag NPs	New Zealand White rabbits	BIC, BA, and BV/TV	Micro-CT and histological evaluation	Control: Ti; test groups: (1) Ag-PIII and (2) Mg/Ag-PIII	8 weeks
Godoy-Gallardoa, 2016 [48]	Implants with silver electrodeposition	Ag electrodeposition	Beagle dogs	BIC and BV/TV	Micro-CT and histological evaluation	Control: Ti; test group: Ti-Ag	2 months
Cheng, 2016 [49]	Silver (Ag)-loaded nanotubular structures	Ag NPs	Rats	BIC, BA, and BV/TV	Micro-CT and histological evaluation	Control: TiO2-NTs; test groups: (1) NT10-Ag1, (2) NT10-Ag2, and (3) NT40-Ag1	6 weeks
Smeets, 2017 [50]	Silver-doped polysiloxane coating	Ag particles	Pigs	BIC	Histological evaluation	Control: grit-blasted and acid-etched Ti implants coated with polysiloxane; test group: silver-doped polysiloxane	3 months
Liu, 2019 [51]	Ti-Ag alloy nanotubular coatings (TNT)	Ag ions	Sprague–Dawley rats	BA	Micro-CT and histological evaluation	Control: TNT; test groups: (1) Ti1% Ag-NT, (2) Ti2% Ag-NT, and (3) Ti4% Ag-NT	(1, 2, and 4 weeks)
Xie, 2019 [52]	Coating consisting of hydroxyapatite (HA), AgNPs, and chitosan (CS)	Ag NPs	SD rats	BIC	Micro-CT and histological evaluation	Control: Ti implants; test group: HA/Ag/CS-coated implants	12 weeks
Stein, 2020 [53]	Titanium–vanadium implants (Ti6Al4V) coated with a polysiloxane layer with embedded elementary silver particles	Ag particles	Merino sheep	BIC	Histological evaluation	Control: uncoated Ti implants; test group: silver-coated implants	6 months
Chen, 2020 [54]	TiO_2_ nanotubes loaded with silver nanoparticles	Ag NPs	Sprague–Dawley rats	BV/TV	Micro-CT analysis	Control: TiO_2_ nanotubes (TiO_2_-NTs); test group: AgTiO2-NTs	2 weeks
Nakashima, 2022 [28]	Silver-containing hydroxyapatite coating	Silver oxide	Sprague–Dawley rats	BIC, BA, and BV/TV	Micro-CT and histological evaluation	Control: HA; test group: Ag-HA	4 and 8 weeks
Yao, 2022 [55]	Magnetron sputtering was used to form uniform Ag and strontium titanate (SrTiO_3_) coatings on Ti	Ag ions	New Zealand White rabbits	BV/TV	Micro-CT analysis	Control: AH-Ti; test groups: (1) AH-Ti/Ag and (2) AH-Ti/Ag/Sr	1 month
Li, 2023 [56]	Ti implants immersed in AgNP solution	Ag NPs	Sprague–Dawley rats	BIC, BA, and BV/TV	Micro-CT and histological evaluation	Control: Ti; test group: AgNP	4 weeks
Pinera-Avellaneda, 2023 [57]	Ti implants with an Ag-doped calcium–Ti surface layer	Ag ions	New Zealand albino rabbits	BV/TV	Micro-CT analysis	Control: Ti; test group: Ti-Ag	2 and 4 weeks

**Table 2:** Quality coefficients of included studies.

Authors	Year	Animal Model	Coefficient	Quality
Cheng et al. [43]	2013	Rat	0.761	Average
Svensson et al. [44]	2013	Rabbit	0.809	Excellent
Qiao et al. [45]	2015	Dog	0.880	Excellent
Devlin-Mullin et al. [46]	2015	Rat	0.785	Average
Zhao et al. [47]	2015	Rabbit	0.714	Average
Godoy-Gallardoa et al. [48]	2016	Dog	0.904	Excellent
Cheng et al. [49]	2016	Rat	0.738	Average
Smeets et al. [50]	2017	Pig	0.904	Excellent
Liu et al. [51]	2019	Rat	0.761	Average
Xie et al. [52]	2019	Rat	0.904	Excellent
Stein et al. [53]	2020	Sheep	0.857	Excellent
Chen et al. [54]	2020	Rat	0.714	Average
Nakashima et al. [28]	2022	Rat	0.928	Excellent
Yao et al. [55]	2022	Rabbit	0.761	Average
Li et al. [56]	2023	Rat	0.857	Excellent
Pinera-Avellaneda et al. [57]	2023	Rabbit	0.761	Average

## Data Availability

No new data were created or analyzed in this study. Data sharing is not applicable to this article.

## Supplementary Materials

The following supporting information can be downloaded at https://www.mdpi.com/article/10.3390/jfb16100369/s1, Table S1: PRISMA checklist. Reference [78] is cited in the Supplementary Materials.

## Abbreviations

The following abbreviations are used in this manuscript:

BIC	bone–implant contact
BA	bone area
BV/TV	bone volume
